# Supplementary material for: Consequences of climate-induced range expansions on multiple ecosystem functions
Source: Commun Biol. 2023 Apr 10;6:390. doi: 10.1038/s42003-023-04673-w (PMC10085988; doi:10.1038/s42003-023-04673-w)
Supplement: Supplementary file 2 — Supplemental Materials [file 42003_2023_4673_MOESM2_ESM.pdf]

Supplemental Materials for:

Consequences of climate-induced range expansions on multiple ecosystem functions

Authors: Jared A. Balik[1,2,3*], Hamish S. Greig[2,4], Brad W. Taylor[1,2], Scott A. Wissinger[2,3]

Affiliations:

[1]Department of Applied Ecology, North Carolina State University; Raleigh, NC 27695, USA

[2]Rocky Mountain Biological Laboratory, Crested Butte, CO 81224, USA

[3]Departments of Biology and Environmental Science, Allegheny College, Meadville, PA 16335, USA

[4]School of Biology and Ecology, University of Maine, Orono, ME 04469, USA

*Corresponding author. Email: Balikj3@gmail.com

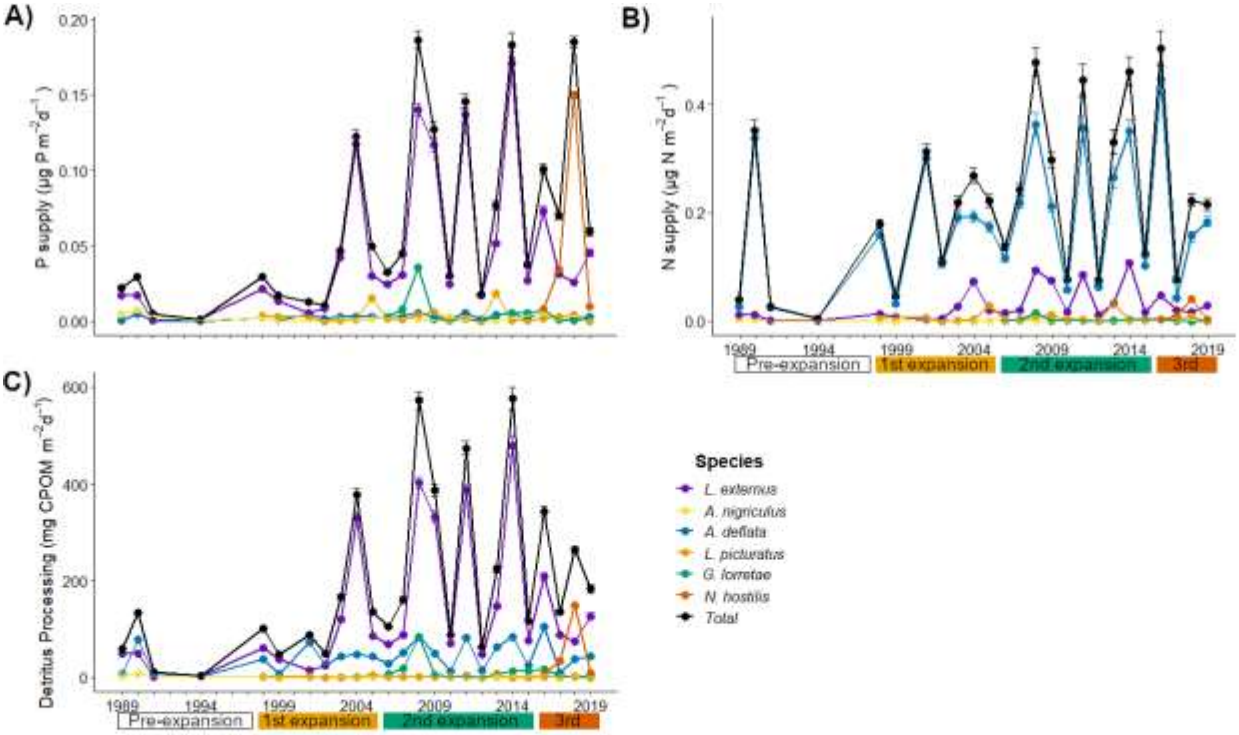

Figure S1. Caddisfly species' predicted areal daily contributions to phosphorus and nitrogen supplies and detritus processing. Predictions are the products of pond-level density, average final instar masses, and species-specific nutrient excretion or detritus processing rates. Intraspecific variation in species-specific rates was incorporated by repeating pond-level calculations 1000 times with randomly sampled species-specific rates in each iteration. Nutrient supply and detritus processing were averaged across all pond-level iterations to estimate the mean ± 1 SE species-specific contributions to P supply [(**A**)], N supply [(**B**)], and detritus processing [(**C**)]. Black points and lines indicate average total caddisfly abundance or total contribution to ecosystem processes.

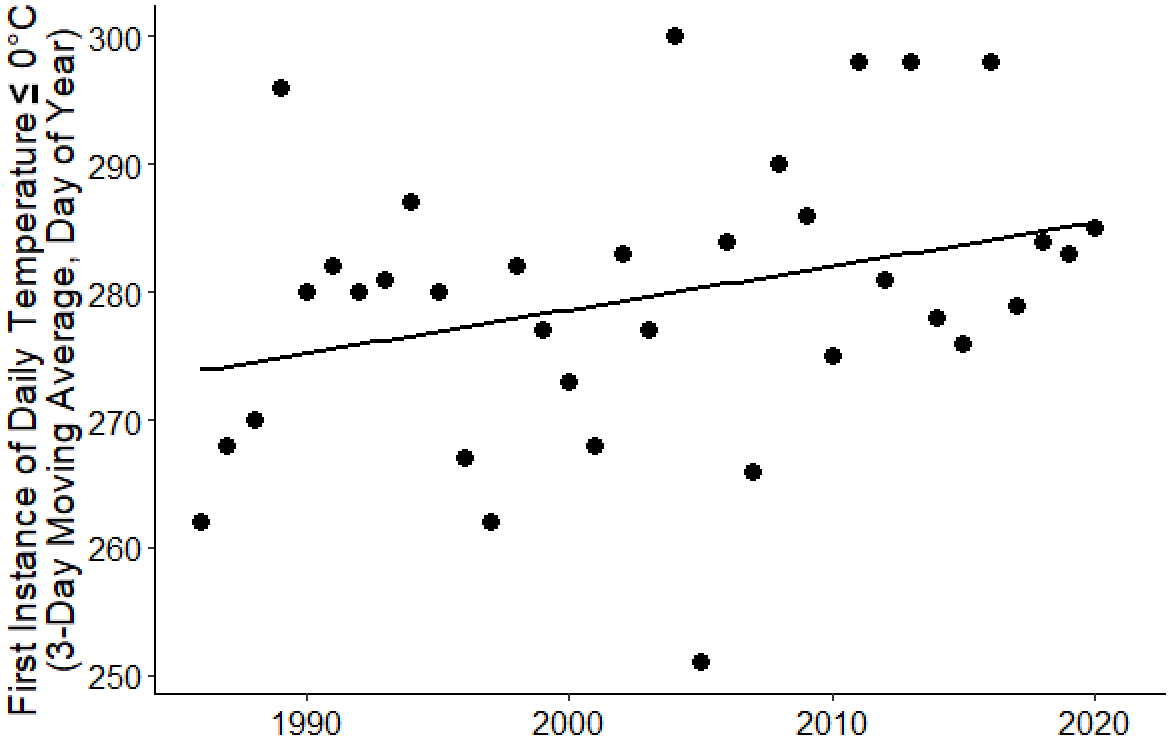

Figure S2. Onset of autumn freeze is occurring later at Schofield Pass (USDA NRCS SNOTEL Site 737). A 3-day moving average daily temperature was used to determine the first date of subfreezing (≤ 0 °*C*) autumn temperature at the Schofield Pass SNOTEL Site 737 (data retrieved from [33]; ($F_{1,33}$ = 3.56, p = 0.068, adj R^2 = 0.07). Relative to the Mexican Cut, this weatherstation is approximately 2 km southeast and 300 m lower in elevation.

term	df (num,den)	F	p
(Intercept)	1,684	1.69	0.195
Year	1,684	1.73	0.189
Species	5,684	1.73	0.126
Range Expansion	3,684	2.30	**0.076**
Year:Species	5,684	1.75	0.122
Year:Range Expansion	3,684	2.31	**0.075**
Species:Range Expansion	15,684	3.25	**<0.001***
Year:Species:Range Expansion	15,684	3.25	**<0.001***

Table S1. Mixed model F test of species relative abundance in permanent ponds. Year, species, range expansion period, and their interactions were modeled as fixed effects. Model also included pond as a random effect and a third-order autocorrelation function (ACF) of year nested within pond. Bold p* denote model term is statistically significant at $\alpha=0.05$, bold only denotes statistically significant at $\alpha=0.10$.

term	P Supply df (num,den)	P Supply F	P Supply p	N Supply df (num,den)	N Supply F	N Supply p	CPOM Processing df (num,den)	CPOM Processing F	CPOM Processing p
(Intercept)	1,339	0.212	0.645	1,339	0.156	0.693	1,339	0.008	0.931
Year	1,339	0.209	0.648	1,339	0.154	0.695	1,339	0.007	0.931
Group	2,339	1.951	0.144	2,339	0.133	0.876	2,339	1.051	0.351
Range Expansion	3,339	2.982	**0.031***	3,339	0.104	0.958	3,339	0.614	0.606
Year:Group	2,339	1.972	0.141	2,339	0.139	0.870	2,339	1.056	0.349
Year:Range Expansion	3,339	2.988	**0.031***	3,339	0.104	0.958	3,339	0.616	0.605
Group:Range Expansion	6,339	5.494	**<0.001***	6,339	1.440	0.199	6,339	3.114	**0.006**
Year:Group:Range Expansion	6,339	5.507	**<0.001***	6,339	1.439	0.199	6,339	3.120	**0.006**

Table S2. Mixed model F tests of species groups' relative contributions to ecosystem processes in permanent ponds. Year, species group (i.e., dominant resident, subdominant resident, range expanding species), range expansion period, and their interactions were modeled as fixed effects. Models also included pond as a random effect, and a third-order autocorrelation function (ACF) of year nested within pond. Bold p* denote model term is statistically significant at α=0.05., bold only denotes statistically significant at α=0.10.

term	P Supply<br>df (num,den)	F	p	N Supply<br>df (num,den)	F	p	CPOM Processing<br>df (num,den)	F	p
(Intercept)	1,109	2.41	0.124	1,109	1.26	0.265	1,109	2.56	0.112
Year	1,109	2.42	0.123	1,109	1.27	0.262	1,109	2.58	0.111
Range Expansion	3,109	0.72	0.541	3,109	1.47	0.227	3,109	0.96	0.413
Year:Range Expansion	3,109	0.72	0.542	3,109	1.47	0.227	3,109	0.96	0.413

Table S3. Mixed model F tests of caddisfly assemblage-total contributions to ecosystem processes in permanent ponds. Year, range expansion period, and their interaction were modeled as fixed effects. Models also included pond as a random effect, and a third-order autocorrelation function (ACF) of year nested within pond. Bold p* denote model term is statistically significant at $\alpha=0.05$.
