# Supplementary material for: Consequences of climate-induced range expansions on multiple ecosystem functions
Source: Commun Biol. 2023 Apr 10;6:390. doi: 10.1038/s42003-023-04673-w (PMC10085988; doi:10.1038/s42003-023-04673-w)
Supplement: Supplementary file 3 — Description of Additional Supplementary Files [file 42003_2023_4673_MOESM3_ESM.pdf]

Description of Additional Supplementary Files

File name: Supplementary Data 1

Description: Zipped folder containing R script for estimating caddisflies' contributions to ecosystem processes over long term community surveys, and data (+ corresponding metadata files) to run the script.
