# Supplementary material for: Consequences of climate-induced range expansions on multiple ecosystem functions
Source: Commun Biol. 2023 Apr 10;6:390. doi: 10.1038/s42003-023-04673-w (PMC10085988; doi:10.1038/s42003-023-04673-w)
Supplement: Supplementary file 5 — Reporting Summary [file 42003_2023_4673_MOESM5_ESM.pdf]

nature portfolio

Corresponding author(s): Jared A. Balik

Last updated by author(s): Jan 6, 2023

Reporting Summary

Nature Portfolio wishes to improve the reproducibility of the work that we publish. This form provides structure for consistency and transparency in reporting. For further information on Nature Portfolio policies, see our Editorial Policies and the Editorial Policy Checklist.

Statistics

For all statistical analyses, confirm that the following items are present in the figure legend, table legend, main text, or Methods section.

n/a	Confirmed	
☐	☒	The exact sample size (n) for each experimental group/condition, given as a discrete number and unit of measurement
☐	☒	A statement on whether measurements were taken from distinct samples or whether the same sample was measured repeatedly
☐	☒	The statistical test(s) used AND whether they are one- or two-sided<br>*Only common tests should be described solely by name; describe more complex techniques in the Methods section.*
☐	☒	A description of all covariates tested
☐	☒	A description of any assumptions or corrections, such as tests of normality and adjustment for multiple comparisons
☐	☒	A full description of the statistical parameters including central tendency (e.g. means) or other basic estimates (e.g. regression coefficient) AND variation (e.g. standard deviation) or associated estimates of uncertainty (e.g. confidence intervals)
☐	☒	For null hypothesis testing, the test statistic (e.g. F, t, r) with confidence intervals, effect sizes, degrees of freedom and P value noted<br>*Give P values as exact values whenever suitable.*
☒	☐	For Bayesian analysis, information on the choice of priors and Markov chain Monte Carlo settings
☒	☐	For hierarchical and complex designs, identification of the appropriate level for tests and full reporting of outcomes
☒	☐	Estimates of effect sizes (e.g. Cohen's d, Pearson's r), indicating how they were calculated

Our web collection on statistics for biologists contains articles on many of the points above.

Software and code

Policy information about availability of computer code

Data collection	All analyses were conducted in R 4.0.2; novel code for predicting caddis assemblage nutrient supply and detritus processing included as electronic supplement.
Data analysis	All analyses were conducted in R 4.0.2; novel code for predicting caddis assemblage nutrient supply and detritus processing included as electronic supplement.

For manuscripts utilizing custom algorithms or software that are central to the research but not yet described in published literature, software must be made available to editors and reviewers. We strongly encourage code deposition in a community repository (e.g. GitHub). See the Nature Portfolio guidelines for submitting code & software for further information.

Data

Policy information about availability of data

All manuscripts must include a data availability statement. This statement should provide the following information, where applicable:

- Accession codes, unique identifiers, or web links for publicly available datasets
- A description of any restrictions on data availability
- For clinical datasets or third party data, please ensure that the statement adheres to our policy

Data included in an electronic supplement.

Human research participants

Policy information about studies involving human research participants and Sex and Gender in Research.

Reporting on sex and gender	N/A
Population characteristics	N/A
Recruitment	N/A
Ethics oversight	N/A

Note that full information on the approval of the study protocol must also be provided in the manuscript.

Field-specific reporting

Please select the one below that is the best fit for your research. If you are not sure, read the appropriate sections before making your selection.

☐ Life sciences ☐ Behavioural & social sciences ☒ Ecological, evolutionary & environmental sciences

For a reference copy of the document with all sections, see nature.com/documents/nr-reporting-summary-flat.pdf

Ecological, evolutionary & environmental sciences study design

All studies must disclose on these points even when the disclosure is negative.

Study description	We combine a 30-year survey of subalpine pond larval caddisfly assemblages with species-specific functional traits (nitrogen and phosphorus excretion, and detritus processing rates) to test how three upslope range expansions affected species' predicted relative contributions to caddisfly-driven nutrient supply and detritus processing. Predicted relative contributions were generated with novel R code included as an electronic supplement. The package "lme4" was used to fit mixed effects models to caddisfly relative abundances, assemblage-total predicted ecosystem processes, and species' relative contributions over time. For mixed models with caddisflies total predicted ecosystem process as the response variable, Year and Range Expansion were modeled as fixed effects along with their interaction. Whereas, when relative contributions or abundance were the response variable, Year, Range Expansion, Group (for relative contributions, i.e., dominant resident, subdominant residents, range expanding species) or Species (for relative abundance), and their interactions were modeled as fixed effects. All mixed models also included Pond as a random effect, and a third-order autocorrelation function (ACF) of year nested within pond. Inclusion of an appropriate ACF order was determined by examining ACF plots for dominant taxon relative abundance; the ACF function for L. externus relative abundance exceeded the α = 0.05 confidence region at lag = 3.
Research sample	Annual census of larval caddisfly populations (five species of Limnephlidae, one of Phryganeidae) at the Mexican Cut Nature Preserve (CO). Annual censuses of pond communities since 1989 indicate that these larval cased caddisflies dominate animal biomass, increasing from 30 to 70 % of animal biomass from permanent to temporary ponds.
Sampling strategy	See "timing and spatial scale" below.
Data collection	See "timing and spatial scale" below.
Timing and spatial scale	Larval caddisflies in lentic habitats were surveyed annually for 25/30 years between 1989 and 2019 with single 0.33 m2 benthic D-net sweeps at the north, east, south, and west sides of 3-7 permanent, 2-11 semi-permanent, and 1-7 temporary ponds. Samples were collected between late June and mid-July each year, approximately 2-3 weeks after pond ice-out. This seasonal timing provides the best annual snapshot of the caddisfly assemblage given their staggered life histories.
Data exclusions	No data were excluded.
Reproducibility	Novel code to generate predicted ecosystem process contributions is provided as an electronic supplement.
Randomization	Larval caddisfly census samples were collected from the same locations (microhabitats) each year for inter-annual consistency; otherwise NA.
Blinding	Not relevant for our study; annual community census.

Did the study involve field work? ☒ Yes ☐ No

Field work, collection and transport

Field conditions	Varied, but sampling generally occurred on clear days for better visibility as all caddisflies were identified to species level in the field.
Location	Mexican Cut Nature Preserve 39°2'N 107°4'W
Access & import/export	The Mexican Cut is a pristine, subalpine (3560 m) wilderness area owned by The Nature Conservancy and managed by the Rocky Mountain Biological Laboratory (RMBL) in the Elk Mountains of central Colorado. All access & import/export was in compliance with RMBL's policies.
Disturbance	NA

Reporting for specific materials, systems and methods

We require information from authors about some types of materials, experimental systems and methods used in many studies. Here, indicate whether each material, system or method listed is relevant to your study. If you are not sure if a list item applies to your research, read the appropriate section before selecting a response.

Materials & experimental systems

n/a	Involved in the study
☒	☐ Antibodies
☒	☐ Eukaryotic cell lines
☒	☐ Palaeontology and archaeology
☒	☐ Animals and other organisms
☒	☐ Clinical data
☒	☐ Dual use research of concern

Methods

n/a	Involved in the study
☒	☐ ChIP-seq
☒	☐ Flow cytometry
☒	☐ MRI-based neuroimaging
